# Ciprofloxacin induced nightmares in an adult patient

**DOI:** 10.4103/0019-5545.44757

**Published:** 2008

**Authors:** Amit Dang, Rajasi Kamat, Rataboli V. Padmanabh

**Affiliations:** Goa Medical College, Bambolim, Goa - 403 202, India. E-mail: dramit_gmc@rediffmail.com; 1 Department of Preventive and Social Medicine, Bambolim, Goa - 403 202, India; 2 Department of Pharmacology, Bambolim, Goa - 403 202, India

Sir,

A 24 year old female, doctor by profession self medicated herself with tablet ciprofloxacin 500 mg BD for 5 days after she was diagnosed to have severe gastroenteritis. Two days later, she reported nightmares which continued for four consecutive days.

On history taking, she could remember two of the frightful experiences that she dreamt of. In one of the dreams, she visualized a lady - whom she had never seen before - who, after calling two girls to her place, brutally assaulted them, slaughtered them by cutting their throats with a knife and crucified them alive. In another dream, she saw herself stranded in a dilapidated haunted house with strange and supernatural things happening around her.

Every time she used to get up with sweating, palpitations, fear, anxiousness, and nervousness. She was irritable and experienced mood changes, while on therapy. The dreams subsided spontaneously after a day of completing her course for 5 days. Causality assessment score by Naranjo Algorithm was 9, putting it in a “highly probable” Adverse Drug Reaction (ADR) category.

Fluoroquinolones are one of the most commonly prescribed antibiotics for urinary tract infections and gastroenteritis and exhibit broad spectrum antimicrobial spectrum.

Fluoroquinolones have always been under controversy due to their serious ADR profile. Temafloxacin and grepafloxacin are already withdrawn from the US market as a result of reported multi system toxicity and torsades de pointes cases, respectively, sparfloxacin for its quinolone toxicity (QT) prolongation and phototoxicity, trovafloxacin has been forbidden in Europe and United Kingdom due to several deaths reported caused by hepatic toxicity and off late Bristol-Myers Squibb Co. announced the withdrawal of their brand of gatifloxacin *(Tequin)* due to dysglycaemia reported in many patients.[1]

Permanent neurological, visual, and musculoskeletal damage has been reported in many patients taking this particular group of antibiotic and most of them are unaware that their illness is a manifestation of QT.

Central Nervous System (CNS) ADRs of fluoroquinolones are well documented. These ADRs can range from patients getting mild confusion to acute psychosis.[2] Rataboli *et al.* reported an unusual hallucination with norfloxacin.[3] The case histories at times can be so perplexing that the doctors refer such patients to the psychiatrists as it is difficult to believe that it could be a drug reaction as well.

The exact pathology of CNS effects is still not understood but one of the hypotheses suggests the stimulatory effect of quinolones to drug interactions with the Gamma-Amino Butyric Acid receptor (GABA), an inhibitory neurotransmitter, and direct activation of N-methyl-D-aspartate (NMDA) and adenosine receptor mechanisms.

Sleep disorders are very rare, but recognized adverse events with fluoroquinolones.[4] Couple of case reports have been published in the past showing fluroquinolone therapy causing agitated sleepwalking and nightmares particularly in the paediatric age group.[5] An episode like the one we report in an adult is very rare and merits a mention.

Nightmares are defined as nocturnal episodes of intense anxiety and fear associated with a vivid, emotionally charged dream experience and is generally classified as parasomnia. Several possible pharmacologic mechanisms for drug-induced nightmares have been proposed as mentioned above. However, the vast majority of therapeutic agents implicated in causing nightmares have no obvious pharmacologic mechanism. Even assessing the causality with such an event is a difficult task because of the high incidence of nightmares in the healthy population as well. In our case, the causality assessment of this ADR was relatively simpler because the nightmares lasted for four days throughout the duration of therapy and disappeared at the end of the course.

One of the reviews published showed sedatives/hypnotics, beta-blockers and amphetamines as the most common therapeutic modalities associated with nightmares.[6] Australian ADR bulletin reported many suspected cases of drug induced nightmares with SSRIs, benzodiazepines and statins.[7]

Hence, we conclude that prescribers especially psychiatrists should be aware of the fact that there are many such drugs that may cause nightmares and other CNS symptoms, which they may mistakenly diagnose as some psychiatric illness. Taking proper history of drug intake may become an important tool to diagnose the same in such a rare scenario.
